# Algorithmic Noise Adjacency in Cantonese-Language Dementia Education on YouTube: Protocol for a Sociotechnical Audit of Recommendation Environments

**DOI:** 10.2196/101949

**Published:** 2026-10-26

**Authors:** Benjamin K P Woo

**Affiliations:** 1 Chinese American Health Promotion Laboratory (CAHPL) University of California, Los Angeles Los Angeles, CA United States

**Keywords:** dementia, digital health, Chinese American, YouTube, algorithms, health equity, aging, social media, infodemiology, protocol

## Abstract

**Background:**

Older Chinese American adults with limited English proficiency frequently rely on culturally and linguistically tailored online media platforms for dementia education and health literacy. Existing digital health evaluation frameworks primarily assess the internal quality and accuracy of educational materials while overlooking the recommendation environments through which users encounter health information. Commercial recommendation systems may expose users to medically unverified or commercially motivated health content adjacent to evidence-based dementia education resources.

**Objective:**

This protocol establishes a reproducible computational framework for auditing recommendation environments surrounding Cantonese-language dementia education videos on YouTube. The study introduces the concept of algorithmic noise adjacency, defined operationally as the concentration of recommendation nodes whose informational utility diverges from evidence-based dementia education objectives within local recommendation neighborhoods.

**Methods:**

The protocol uses an automated sociotechnical audit framework centered on 2 Cantonese-language dementia education videos previously examined in longitudinal digital outreach research. A Selenium WebDriver pipeline with a headless Chromium browser architecture and fingerprinting mitigations will simulate 1200 independent browsing sessions distributed uniformly across a 100-day window. Sessions will be routed through a rotating residential proxy infrastructure localized to Southern California Chinese American communities. During each session, the top 10 sidebar recommendation videos adjacent to the source clinical asset (the anchor video) will be extracted. Recommendation metadata will undergo structured semantic classification into 5 mutually exclusive categories: category A, verified public health and clinical infrastructure, category B, diaspora culture and entertainment, category C, commercially motivated health content lacking established clinical validation, category D, unverified alternative medicine, and category E, ambiguous or unclassified baseline noise. Double-coding and consensus adjudication of a random 10% sample will be used to establish interrater reliability. Generalized linear mixed-effects models with logit link functions, session-level random intercepts, and reciprocal rank slot position weighting will evaluate recommendation characteristics while accounting for clustering within browsing sessions. Primary outcome measures include the noise adjacency ratio, slot-weighted noise adjacency ratio, recommendation recurrence density, and neighborhood entropy. Secondary analyses will evaluate temporal recommendation drift and cross-session recommendation variability.

**Results:**

Protocol development and pilot automation testing were finalized in September 2026. Automation stress testing, semantic calibration, and proxy validation are scheduled for January 2027. Data collection is projected to occur over a 100-day interval following deployment of the finalized extraction architecture.

**Conclusions:**

This protocol proposes a structural informatic auditing framework for minority-language digital health ecosystems. By shifting evaluation from isolated content quality toward surrounding recommendation neighborhoods, the study may provide digital health researchers with a reproducible methodology for characterizing health information exposure conditions among linguistically isolated populations.

**International Registered Report Identifier (IRRID):**

PRR1-10.2196/101949

## Introduction

### Background

Digital media platforms increasingly function as health communication infrastructures for older adults with limited English proficiency. YouTube has also been examined as a medium for disseminating psychoeducation, supporting its potential role as an accessible channel for mental health communication [1]. Among Chinese American populations, culturally and linguistically tailored dementia education distributed through platforms such as YouTube and WhatsApp has demonstrated sustained engagement and educational utility [2]. Community-based evidence from Cantonese-language health broadcasting further illustrates the importance of culturally and linguistically concordant communication infrastructures for reaching linguistically isolated immigrant populations [3]. Prior longitudinal work has shown that culturally concordant digital outreach can reduce dementia stigma and improve access to care among older Chinese American adults [4]. Prior work has also demonstrated the feasibility of using digital media platforms to reach older Chinese American adults for health promotion and fall prevention messaging [5]. This intersection of digital communication pathways and public health monitoring defines the emerging field of infodemiology [6], which is becoming vital for scaling digital health interventions and hybrid models of psychiatric care to underserved populations [7,8].

Despite increasing attention to digital health equity and systemic algorithmic bias within public frameworks [9,10], most digital health evaluation frameworks focus primarily on the internal characteristics of educational materials, including readability, source credibility, and informational accuracy [11]. Recent evaluation of YouTube videos for family caregivers of people living with Alzheimer disease dementia similarly illustrates the emphasis placed on the quality and reliability of individual videos [11]. Comparatively less attention has been directed toward the recommendation environments through which users encounter health information online. Empirical studies of major social platforms have shown that algorithmic ranking and filtering substantially shape the information users encounter [12]. More recent experimental evidence indicates that feed algorithms can also influence attitudes and behavior [13]. While the present protocol does not examine behavioral or attitudinal outcomes, these findings underscore why the composition of the local recommendation neighborhood surrounding evidence-based health content is itself a relevant object of measurement. Recent work on YouTube health communication has similarly emphasized that clinically accurate, expert-produced content may have limited visibility and engagement, highlighting the importance of examining platform-level dissemination rather than content quality alone [14].

Commercial recommendation systems optimize engagement-oriented objectives rather than clinical information objectives [15]. Consequently, users viewing evidence-based dementia education materials may simultaneously encounter adjacent recommendation nodes promoting medically unverified interventions, commercially marketed wellness products, or non–evidence-based health claims [16]. However, the presence of particular types of content on a platform should not be assumed to represent actual exposure among individual users. For older adults with limited English proficiency, inconsistent recommendation environments may complicate dementia literacy navigation and increase exposure to conflicting health information. Although adjacent unverified or commercial content may shape the surrounding information environment, this study measures only structural adjacency and content composition; it does not infer user exposure, persuasion, propagation, or behavioral effects.

### Conceptual Framework

This study introduces the concept of algorithmic noise adjacency, defined as the concentration of recommendation nodes whose informational utility diverges from evidence-based dementia education objectives within local recommendation neighborhoods.

Rather than viewing a video as an isolated educational asset, this protocol conceptualizes the recommendation environment as a sociotechnical information network. In this network, the selected dementia education video serves as the anchor video, and the surrounding sidebar elements constitute a local recommendation neighborhood. This neighborhood is not random; it is shaped by a mix of YouTube’s baseline platform architecture, local geographic routing, and real-time feed variations. Rather than attempting to reverse-engineer or mathematically model the proprietary backend platform algorithms, this protocol establishes an empirical, purely observational methodology to classify and quantify these structural properties as they appear to the end user.

### Study Objectives

The primary objective is to establish a reproducible computational framework for auditing recommendation environments surrounding Cantonese-language dementia education videos on YouTube [17]. In particular, this study will use 2 specific Cantonese-language dementia education videos under a standardized, repeated audit configuration to characterize the recommendation neighborhoods.

Secondary objectives are (1) to quantify the prevalence of medically unverified recommendation nodes, (2) to evaluate positional differences within recommendation hierarchies, (3) to assess whether multilingual metadata density influences recommendation quality, (4) to characterize recommendation recurrence structures and neighborhood entropy, and (5) to develop a reproducible methodology for future minority-language algorithmic audits.

The study does not aim to estimate population-level human exposure, the prevalence of potentially disruptive recommendations across YouTube, or the recommendation environment experienced by Cantonese-speaking populations generally. Generalization beyond the 2 audited assets is outside the scope of the current study.

## Methods

### Study Design

This study is a computational sociotechnical audit of publicly accessible recommendation environments associated with minority-language dementia education videos hosted on YouTube (Alphabet Inc) [15,17]. Repeated automated browsing sessions will simulate standardized viewing conditions while minimizing contamination from persistent browser history, cookies, or individualized personalization artifacts. The protocol will be preregistered on the Open Science Framework before initiation of data collection. Because this study is a computational sociotechnical audit protocol rather than a clinical trial or systematic literature review, no single established reporting guideline maps exactly to the study design; the protocol was prepared with reference to applicable protocol-reporting principles and relevant guidance for observational audits.

### Asset Selection

Two Cantonese-language dementia education videos originally published in January 2014 were selected because of their longitudinal platform stability, continued public accessibility, and prior use in dementia outreach research involving older Chinese American populations [2,4]. The 2 anchor videos, from the same research-associated channel, were selected as a bounded case study pair rather than as a statistically representative sample of Cantonese-language dementia education videos. The first asset, Understanding Dementia (認識腦退化症 (老年癡呆症 or 老人癡呆症) [18], focuses on dementia psychoeducation and stigma reduction. The second asset, Dementia Prevention and Management (防治腦退化 (防治老人痴呆症) [19], focuses on dementia prevention strategies, behavioral interventions, and health care navigation.

English-translated titles are used throughout the manuscript to improve indexing consistency and editorial readability.

### Automation Architecture

Automated browsing sessions will be conducted using a custom Python (version 3.10; Python Software Foundation) script operating Selenium WebDriver (version 4.20 or higher) within a headless Chromium (version 124 or higher) browser environment. Headless execution modes introduce known browser fingerprinting discrepancies (eg, the absence of WebGL vendor strings or distinct plugin arrays) that automated web systems use to identify nonhuman traffic. To ensure behavioral equivalence with standard user clients, the pipeline integrates the *selenium-stealth* package to override the automated webdriver property, apply standard hardware acceleration variables, and inject a consistent desktop user-agent string representing a standard Windows-based Google Chrome user. The automated browsing pipeline does not bypass authentication, CAPTCHAs, access controls, rate limits, robot directives, or other platform safeguards, and data collection will comply with applicable platform requirements.

Each individual browsing session executes the following discrete sequence: (1) initialization of a clean browser container within a completely unique, temporary profile directory, ensuring explicit suppression of persistent cookies, local storage, and session cache; (2) routing through rotating residential proxy infrastructure localized to Southern California zip codes with dense (>25%) Chinese American immigrant populations; (3) direct navigation to the target YouTube URL; (4) maintenance of an active session for 180 seconds (we settled on this duration after a 50-session pilot test showed that the sidebar recommendations consistently loaded and stabilized within 45 seconds of the initial page render); (5) extraction of the text and metadata of the top 10 sidebar recommendation videos from the rendered Document Object Model; and (6) destruction of the browser instance, deletion of the temporary profile directory, and forced proxy IP rotation.

The automation architecture is designed solely for observational metadata analysis and performs no platform manipulation, engagement fabrication, or user interaction. To ensure full compliance with platform guidelines and broader ethical web crawling practices, the architecture enforces a strict rate limit (maximum of 1 request per 2 minutes per proxy IP) to avoid imposing an artificial service load, respects automated server-side responses, and restricts access strictly to publicly accessible metadata elements without circumventing authentication layers.

### Data Extraction

For each individual recommendation video extracted, the following metadata fields will be recorded in a secure SQL database: recommendation video unique ID and title text; source channel identifier, channel description, and official verification status; upload date and total view count; video duration in seconds; and sidebar slot position index (numbered 1 through 10, sequentially from top to bottom). No user accounts, login credentials, private communications, or identifiable personal information will be accessed or stored.

### Sampling Framework and Sizing

To capture diurnal, weekly, and seasonal algorithmic variations, each source asset will undergo 600 independent browsing sessions distributed uniformly at randomized intervals (approximately 6 sessions per day) across a continuous 100-day window. While the total platform vocabulary of unique videos is vast, our observation window is strictly bounded to the top 10 recommended sidebar elements presented during each session. A total of 600 sessions per asset were selected to provide repeated observations across the 100-day audit period and to characterize temporal variability. Because clustering and recurrence reduce the effective sample size, this study is considered an exposure-mapping audit rather than a formally powered hypothesis test. The total structural dataset size will consist of 12,000 total recommendation nodes (2 assets multiplied by 600 sessions multiplied by 10 sidebar slots). The nominal number of recommendation slot observations should not be interpreted as the number of independent statistical observations. Recommendation slots within the same session share the same anchor, browsing configuration, time point, and session-level conditions. In addition, the same recommendation video identifiers may recur across sessions, producing dependence across observations. The 2 anchor assets are also fixed case study units rather than a random sample from a larger population of videos.

### Semantic Classification Framework

#### Overview

Extracted recommendation nodes will be classified into 5 mutually exclusive categories using a prespecified rule-based classifier operating on the video title, description, channel name and description, outbound links, verification status, and other recorded metadata. The classifier will be frozen prior to data collection. A hierarchical decision procedure based on video title text, channel name and description, available video description text, channel-level metadata, platform verification indicators, and links or other metadata that indicate commercial activity will be implemented. Classification will be performed using language-specific lexical dictionaries and rule-based feature matching, followed by the prespecified rule-based classifier.

The framework is designed to distinguish source credibility, culturally normative content, commercially motivated health claims, and unverified alternative medicine claims rather than to treat all nonclinical content as noise.

#### Category A: Verified Public Health and Clinical Infrastructure

This category includes videos originating from channels possessing YouTube’s official health verification badge or explicitly matched against a whitelist of academic medical centers, governmental health agencies (eg, the Centers for Disease Control and Prevention or the World Health Organization), and licensed health care networks.

#### Category B: Diaspora Culture and Entertainment

This category encompasses culturally normative, nonmedical content, including Cantonese-language sitcoms, cooking programs, regional news broadcasts, and lifestyle programming (eg, TVB dramas, Cantonese cooking channels, regional news, and lifestyle vlogs).

#### Category C: Commercially Motivated Health Content Lacking Established Clinical Validation

This category includes videos whose metadata and channel description keywords indicate a health-related claim concerning dementia, cognition, memory, prevention, treatment, or related health outcomes, and at least one prespecified commercial signal is present, while the claimed intervention cannot be established from the available evidence and source information as an evidence-based clinical intervention. Commercial signals may include title structures and links to commercial e-commerce storefronts selling brain supplements, affiliate or sales language, product names associated with commercial offerings, explicit calls to purchase, or channel-level descriptions indicating monetization of the promoted health intervention. Lexical matching alone will not be sufficient for category C assignment when the surrounding metadata do not establish a direct commercial health claim.

#### Category D: Unverified Alternative Medicine

This category includes videos promoting pseudoscientific diagnostic frameworks, non–evidence-based dementia treatments, or mystical or supernatural therapeutic claims lacking peer-reviewed clinical data (eg, an anonymized channel labeled *Frequency-Healing-Dementia-Cure*). Category D does not require evidence of commercial monetization.

#### Category E: Ambiguous or Unclassified Baseline Noise

This category covers content that cannot be confidently mapped to categories A through D or items exhibiting multilabel features that fail automated keyword thresholds. Category E represents classification uncertainty rather than a substantive content type. Sensitivity analyses excluding category E will assess the effect of this choice.

### Language Handling and Lexical Resources

Because the audit concerns Cantonese-language and Chinese-language recommendation environments, classification will use language-specific dictionaries rather than relying on English-language keywords alone. Text will be normalized using Unicode normalization, traditional-to-simplified character mapping for matching purposes while retaining the original text, and detection of Chinese-English code-switching. The lexicon will include Cantonese and written-Chinese expressions, common Cantonese variants, transliterated product and treatment names, and relevant English terms. Literal machine translation alone will not be used as the classification rule.

### Classification Reliability and Automated Classifier Validation

Automated classification will be treated as a measurement instrument and will be independently validated against a human-adjudicated reference standard before primary data collection. Human interrater reliability and automated classifier performance will be evaluated as separate components of classification validity. During the pilot phase, a prespecified validation sample of recommendation nodes will be drawn from the same sampling frame and acquisition procedure planned for the primary study. The validation sample will be stratified to ensure representation of all 5 categories, including categories C and D, category E cases, and cases containing potentially ambiguous or conflicting classification indicators.

Two bilingual human reviewers trained on a standardized coding manual will independently classify each validation node without access to the automated classifier’s assigned category. Reviewers will assess the same metadata fields available to the automated classifier. Disagreements will be resolved by a senior reviewer. The consensus label will constitute the reference standard classification.

Human-human agreement will be assessed separately using Cohen κ, with a prespecified minimum value of 0.80 for the coding process. Agreement between the automated classifier and the reference standard will be reported for each category using a confusion matrix, sensitivity, specificity, positive predictive value, negative predictive value, accuracy, and 95% CIs. Because the primary outcome combines categories C and D, performance will also be reported for C and D vs not C and D, including sensitivity, specificity, positive predictive value, and negative predictive value.

The classifier rules and lexicon will be frozen before validation and will not be modified using the validation labels. Any modification prompted by validation will be documented as a protocol amendment, followed by repeat validation on a newly selected sample.

### Primary Outcome Measures

#### Noise Adjacency Ratio

The unweighted noise adjacency ratio (NAR) measures the simple proportion of potentially disruptive health information nodes within an individual browsing session. It is operationally defined as the total number of category C and category D videos observed in the sidebar, divided by the total number of recommendation slots extracted per session (which is fixed at 10). For the primary outcome, a recommendation node will be coded as exposed if the validated classification is category C or D. Automated labels will be treated as measurement outputs subject to the validation and misclassification analyses described below, rather than as error-free observations.

#### Slot-Weighted NAR

Upper-slot recommendation nodes receive higher exposure weights than lower-slot nodes to account for the decaying visual salience across recommendation positions on desktop layouts [20]. The primary slot-weighted NAR uses reciprocal-rank weights *w_r_*=1/*r*, selected to give progressively greater weight to earlier slots. As a robustness analysis, we will also use the logarithmic discount *w_r_*=1/log_2_(*r*+1). The weighting scheme will not affect the unweighted NAR. Using a reciprocal-rank discount model, the position weight for any given slot is defined as 1 divided by its slot position index (resulting in a weight of 1.0 for position 1, 0.5 for position 2, down to 0.1 for position 10). The normalized slot-weighted NAR for a session is calculated by taking the sum of the position weights of all sidebar slots occupied by category C or category D videos and dividing that value by the total sum of the position weights for all 10 positions.

#### Recommendation Recurrence Density

To distinguish structured platform targeting from transient stochastic noise across the 600 sessions per asset, the recommendation recurrence density evaluates how frequently specific videos reappear. It is defined as the total number of independent browsing sessions in which a unique recommended video ID appears, divided by the total number of completed sessions (600) for that specific anchor asset.

#### Neighborhood Entropy

The structural diversity and concentration of the local recommendation neighborhood within an individual session will be evaluated using standard Shannon entropy. This metric is calculated based on the empirical proportions of the 5 semantic categories (A through E) present within that specific session’s 10 sidebar slots. To ensure mathematical continuity and address categories that do not appear in a given session, any category with a proportion of 0 is explicitly assigned an entropy contribution value of 0. Lower entropy values denote highly concentrated, homogeneous algorithmic targeting, whereas higher entropy indicates a diverse mix of surrounding content.

### Alignment of Outcomes With Study Objectives

The prespecified outcomes map directly onto the study objectives. NAR and slot-weighted NAR address the prevalence and positional distribution of potentially disruptive recommendations, recurrence density and neighborhood entropy characterize recommendation recurrence and neighborhood composition, multilingual metadata characteristics will be evaluated as exploratory predictors of recommendation classification, and temporal drift analyses assess variation across the audit period. The overall protocol architecture addresses the secondary objective of establishing a reproducible audit methodology.

### Statistical Analysis

The primary analysis will use the prespecified automated classifications. Classification error will be evaluated in a prespecified sensitivity analysis using the validation-sample estimates of sensitivity and specificity for the binary C and D outcome. Corrected estimates of C and D prevalence will be calculated using the Rogan-Gladen estimator, where applicable, and uncertainty will be propagated through bootstrap resampling of the validation sample. NAR and slot-weighted NAR will also be recalculated using the adjudicated labels available in the validation sample. For recurrence density and neighborhood entropy, analyses will be repeated under alternative plausible misclassification scenarios based on the observed validation performance, including optimistic, observed, and conservative sensitivity and specificity values. If the lower 95% confidence bound for sensitivity or positive predictive value for C and D is below 0.80, classifier-derived outcome estimates will be labeled exploratory and interpreted with appropriate caution.

Descriptive statistics will summarize the distribution of recommendation categories across all sessions and slot positions. To model the primary outcome—the binary exposure status of an individual recommendation slot (defined as 1 if the video belongs to category C or D and 0 otherwise)—we will use a generalized linear mixed-effects model using a logit link function. The model describes within-case and between-case differences; it does not establish population-level anchor effects.

The model will include fixed effects for the slot position index (1 through 10), the source anchor asset, and the categorical time of day (divided into morning, afternoon, evening, and night blocks) to account for variations in platform behavior. A session-level random intercept will be included to account for clustering within independent browsing sessions.

Secondary longitudinal analyses evaluating temporal drift across the 100-day window will incorporate continuous time predictors using a cubic spline basis within the mixed model to capture nonlinear platform adjustments over time. To control the type I error rate across multiple exploratory secondary end points, a Benjamini-Hochberg false discovery rate correction will be applied. All metrics represent observational exposure mappings; no causal behavioral inferences regarding user interactions or direct clinical harms will be made.

### Ethical Considerations

This study analyzes publicly accessible platform metadata generated through automated observational procedures. The protocol does not involve human participants, patient records, private communications, or identifiable personal information. The study characterizes observable recommendation adjacency structures rather than downstream behavioral outcomes or platform intent. Before data collection, this protocol will be submitted to the UCLA Office of the Human Research Protection Program for a formal determination of whether the project constitutes human subjects research or qualifies for an exemption [21]. No data collection or analysis will begin until the determination is received. The study uses publicly available YouTube content, involves no interaction with users, and will retain only deidentified channel-level and video-level data. The determination reference will be reported in the final manuscript.

## Results

Protocol architecture and computational workflows were finalized in September 2026. Automation stress testing, multilingual semantic calibration, and proxy validation procedures are scheduled for January 2027. Data collection will proceed continuously across a planned 100-day interval following deployment of the finalized extraction pipeline.

The completed dataset will characterize the baseline distribution of recommendation categories (A, B, C, D, and E) in minority-language spaces, slot-level recommendation variation and decay patterns, temporal changes in unweighted and slot-weighted NAR measurements, prevalence and structural density of medically unverified recommendation clustering, and recommendation recurrence structures and neighborhood entropy patterns.

## Discussion

### Principal Results

This protocol proposes a reproducible framework for auditing recommendation neighborhoods surrounding minority-language dementia education media. Rather than evaluating educational videos as isolated informational units, the study conceptualizes recommendation environments as observable exposure topologies within dynamic social media ecosystems [17]. This distinction is consistent with recent YouTube health communication research showing that expert-produced content may achieve high clinical quality while receiving comparatively limited visibility or engagement [14]. The protocol does not infer intentional platform behavior or downstream clinical causality. Instead, it characterizes measurable recommendation adjacency structures surrounding public health media assets. The eventual findings should be interpreted as a bounded case study of 2 Cantonese-language dementia education assets rather than as population-level estimates of YouTube recommendation behavior or human exposure among Cantonese-speaking populations. The principal contribution is therefore methodological rather than prevalence estimating.

Although the present protocol specifies a fully automated, high-volume Selenium-based audit pipeline, the core conceptual framework—algorithmic noise adjacency, the 5-category semantic classification scheme (A-E), the NAR, and the interpretation of local recommendation neighborhoods—can be applied at a smaller scale using simpler observational methods. Independent investigators or trainees who elect to capture screenshots of the YouTube sidebar and manually tally the top 10 recommended videos according to the same category definitions may still reference this protocol as the methodological foundation for their work. In such cases, the automated procedures described herein serve as the reference standard against which manual implementations can be compared, while the operational definitions of noise adjacency and the classification rules remain directly usable. This modularity is intentional: the protocol is designed not only for large-scale reproducible audits but also to support transparent, citable basic observational studies that adopt its conceptual and classificatory architecture.

### Comparison With Prior Work

Previous studies involving Chinese American dementia education primarily focused on engagement metrics, dissemination pathways, and culturally tailored outreach effectiveness [2,4]. This protocol extends this literature by evaluating the recommendation ecosystem itself as an independent object of analysis. This approach aligns with broader algorithmic auditing scholarship, demonstrating that recommendation architectures and automated profiling may shape health information exposure conditions independently of the internal features of the original educational intervention [9,10,15].

Beyond methodological characterization, this framework may have implications for digital health literacy and public health communication. Measuring the informational composition surrounding a clinically oriented video may help distinguish the quality of an individual educational asset from the quality of the broader information environment in which it is encountered. This distinction may be relevant to health information quality assessment, digital literacy initiatives, and future platform governance or public health monitoring efforts, particularly for linguistically isolated populations. However, because the proposed audit measures recommendation adjacency rather than individualized exposure, persuasion, or clinical outcomes, these potential applications should be regarded as hypothesis generating rather than evidence of direct public health effects.

### Limitations

Several limitations should be acknowledged. First, recommendation systems are proprietary and continuously evolving, meaning that findings represent a time-bounded audit of platform behavior during the sampling interval. Second, automated semantic classification may incompletely identify covert advertising structures or nuanced misinformation. Third, although browser-isolation procedures reduce personalization artifacts, recommendation variability cannot be fully eliminated. Fourth, this protocol focuses specifically on 2 Cantonese-language dementia education environments on YouTube and may not generalize to other linguistic or disease-specific ecosystems. Fifth, the protocol evaluates informational exposure environments rather than direct behavioral or clinical outcomes. Finally, the study includes only 2 anchor videos originating from the same channel.

Furthermore, this study measures NAR, which represents content prevalence rather than personalized user exposure. Previous studies have highlighted that content prevalence and actual user exposure via personalization differ substantially, and future work should incorporate engagement-weighted exposure denominators to align reported metrics with real-world exposure [4,12,13].

Last but not least, category E represents nodes that could not be confidently assigned to a substantive category and may therefore reflect measurement uncertainty rather than genuine informational diversity. Including category E in the primary neighborhood entropy calculation could inflate or reduce apparent diversity depending on its distribution. We will address this limitation by reporting a sensitivity analysis in which category E is treated as missing and entropy is recalculated over categories A to D only. Accordingly, entropy estimates should be interpreted as descriptive measures of observed classification heterogeneity rather than definitive measures of informational diversity.

Transferability should consequently be evaluated through replication rather than assumed from the present 2-asset study. Future studies can apply the same outcome definitions and audit procedures to systematically sampled anchor sets and compare the resulting distributions across languages, health topics, channels, geographic configurations, and platform surfaces.

### Conclusions

This protocol introduces a structural informatic auditing framework for minority-language digital health ecosystems using 2 Cantonese-language dementia education assets [18,19]. If validated, the methodology may provide researchers with a reproducible approach for evaluating how recommendation systems influence exposure to medically reliable information among linguistically isolated populations. The framework may additionally inform future efforts to develop culturally concordant and clinically reliable digital public health infrastructures for aging minority communities.

The resulting estimates will describe the recommendation environments associated with these 2 case study assets and will not be interpreted as population-level measures of human exposure, Cantonese-language YouTube content, or minority-language digital health ecosystems. The principal contribution is the development and empirical application of a reproducible measurement framework that can subsequently be replicated with broader and independently sampled anchor sets.

## Abbreviations

NAR: noise adjacency ratio

## Data Availability

The complete replication package, including the automated Python execution scripts, exact Selenium and Chromium configurations, anonymized keyword-matching lexicons, and analytic code, will be made openly available upon study completion under an open-source MIT License via the study’s Open Science Framework repository. Specific residential proxy credentials and precise localized IP metadata will be redacted to preserve operational security and privacy boundaries.
